# MCM6 versus Ki-67 in diagnosis of luminal molecular subtypes of breast cancers

**DOI:** 10.1186/s13000-022-01209-4

**Published:** 2022-02-06

**Authors:** Dorsay Sadeghian, Hana Saffar, Pouya Mahdavi Sharif, Vahid Soleimani, Behnaz Jahanbin

**Affiliations:** 1 Cancer Institute, Pathology Department, Imam Khomeini Hospital Complex, Tehran University of Medical Sciences, Tehran, Iran; 2 Present address: Center for Individualized Medicine-Biomarker Discovery, Mayo Clinic, Rochester, MN USA; 3 School of Medicine, Tehran University of Medical Sciences, Tehran, Iran; 4 Cancer Immunology Project (CIP), Universal Scientific Education and Research Network (USERN), Tehran, Iran

**Keywords:** Breast cancer, MCM6, Ki67, Biomarkers, Luminal a, Luminal B, Diagnostic

## Abstract

**Background:**

Currently, breast cancers are divided into four major molecular subtypes. The distinction between the luminal A and luminal B subtypes is mainly based on the cellular proliferation indices and is assessed by the Ki-67 scoring. Due to the limitations in the assessment and expression of Ki-67, we hypothesized that minichromosome maintenance protein 6 (MCM6) might be taken as a surrogate marker to differentiate molecular subtypes and aid in more precise grading of tumors.

**Methods:**

We performed a retrospective, cross-sectional study on 124 samples of breast cancer and 40 samples of normal breast tissue. Relevant clinical information was retrieved from the Cancer Institute database.

**Results:**

MCM6 could discriminate between various categories of histologic grades, tubule formation, mitotic indices, and nuclear pleomorphism (*P* = 0.002 for tubule formation and *P* < 0.001 for other). Moreover, the MCM6 score exhibited a significant correlation with the mitotic count (*P* < 0.001). However, the Ki-67 score could not discriminate subgroups of the mitotic index and nuclear pleomorphism. Compared to the luminal A subtype, luminal B exhibited a higher MCM6 score (*P* = 0.01). Besides, MCM6 scores were higher for certain subtypes with more aggressive behaviors, such as hormone receptor (HR)-negative disease, and human epidermal growth factor receptor 2 (HER2)-enriched and triple-negative breast cancers, as there was a significantly higher MCM6 mean score in the HR-negative in comparison to the luminal breast cancers (*P* < 0.001). Similarly, higher MCM6 scores were observed among samples with more advanced nuclear grades, tubule formation, and overall grades.

**Conclusion:**

MCM6 can differentiate luminal A and luminal B subtypes and is correlated with mitotic counts. However, this study was unable to prove the superiority of MCM6 in differentiating between molecular subtypes compared to the Ki-67 score. Nevertheless, in our study, MCM6 was superior to Ki-67 in exhibiting correlations with the mitotic grade, tubule formation, and nuclear grades. More studies are needed to standardize its assessment methods, determine more robust cut-off values, and evaluate its associations with prognostic features of breast cancer.

## Background

Breast cancer is the most common neoplasm among women all over the world, and consequently, is associated with considerable morbidity and mortality [1]. Breast cancers are complex tumors with a great spectrum of clinical and pathological features and diverse responses to available treatments [2]. Most treatment modalities are selected according to the molecular subtypes of breast cancers, which incorporate hormone receptors (HR), human epidermal growth factor receptor 2 (HER2), and Ki-67 expression status [3].

The minichromosome maintenance proteins (MCMs) play central roles in many aspects of genomic stability. They are one of the regulatory components of DNA replication and ensure that DNA replicates only once in each cell cycle. MCM family is a hexamer of six related peptides (MCM2–7), and each subunit has distinct functions in the regulation of DNA replication. In a process known as DNA replication licensing, it primes chromatin for DNA replication. It also works as a DNA helicase during the elongation phase of DNA replication [4, 5]. Cells express MCM as they enter the G1 phase, before being involved in the active replication. As a result, they can be identified at elevated levels in no-cycling cells that have proliferative potentials and precancerous cells (but not in quiescent somatic cells). Therefore, they can be taken as a marker for early cancer diagnosis [6–8]. Besides, the essential roles of MCM proteins in the proliferation process make them appropriate targets for anti-cancer agents.

Ki-67 is one of the most well-evaluated proliferation markers in tumor cells (including breast cancer), with diagnostic and prognostic roles. In comparison with MCM proteins, some limitations exist for Ki-67. For instance, Ki-67 is not expressed at the G0 and early G1 phases, which will result in the misidentification of a fraction of tumoral cells at these phases. The exact functions of Ki-67 are not elucidated yet, and prominent variability in its scoring and poor analytical validity is reported [6].

Some studies have evaluated the expression of various members of the MCM proteins in neoplasms of different organs. For instance, it is evident that the MCM2 expression correlates with the malignant status and regulates the proliferation and cell cycle of lung squamous cell carcinoma [9], renal cell carcinoma [10], prostate cancer [11], breast cancers [12], brain tumors [13], lymphoma [14], and gastrointestinal tumors [15, 16]. Another study [17] has investigated the putative diagnostic and prognostic features of MCM2, MCM4, and MCM6 in breast cancer and has demonstrated that all three of them can discriminate between luminal A and B subtypes and are associated with higher histological grades and more aggressive subtypes (including luminal B, HER2-positive, and triple-negative breast cancers [TNBC]) [17].

However, the expression status of MCM proteins (either alone or in combination with other markers) is not widely investigated in breast tumors, and their associations with prognostic indicators, including survival, regional recurrence, and distant metastases, are not evaluated comprehensively [18]. Due to the importance of proliferative markers in the prediction of prognosis of breast cancers, as well as a clinical need for effective targeted therapies (especially for those with more aggressive phenotypes), and based on the reports of a previous study [17], we selected the MCM family protein six (MCM6) and compared it with the Ki-67 scores regarding associations with the histologic types, molecular subtypes, and biomarker status of breast cancer.

## Methods

### Patient selection and sample preparation

In this retrospective, cross-sectional study, we evaluated 124 female patients with breast cancer who had undergone mastectomy or lumpectomy without neoadjuvant therapy between 2007 to 2014. In addition, we selected 40 normal breast tissues sample of women who had undergone plastic surgery (reduction mammoplasty) as the control group.

The best block of formalin-fixed paraffin-embedded samples (FFPE) of cases were selected for immunohistochemistry (IHC) staining and evaluated independently by two pathologists for grading according to the Elston-Ellis modification of Scarff-Bloom-Richardson grading system, also known as the Nottingham grading system [19, 20]. This grading system evaluates three morphological pathologic features, including tubule formation, nuclear pleomorphism, and mitotic count, and based on the sum of scores for each feature establishes an overall histologic grade [19, 20]. We selected the best-preserved sites of tumors with the highest mitotic figures, acquired 4 mm punches, and prepared new tissue array paraffin blocks.

For control group samples, we selected one tissue block and completely sectioned it for H&E and IHC staining to determine the status of proliferative markers. All procedures and the data retrieval were conducted after the approval of the Tehran University of Medical Science research ethical committee (ethics code: IR.TUMS.IKHC.REC.1397.113).

### Immunohistochemistry

IHC assay of the newly prepared FFPE slides of tumors and selected paraffin blocks of normal breast tissues of the control group was performed manually. IHC staining was performed using the following antibodies: MCM6 (recombinant rabbit monoclonal antibody, clone number EPR17686, ab201683, 1/10000 dilution), followed by goat anti-rabbit IgG H&L (HRP, ab97051, 1/500 dilution), TRIS EDTA, and Ki-67 (rabbit IgG anti-human Ki-67 monoclonal antibody, clone SP6, 1/50 dilution, Master Diagnostica).

Slides were examined, and the best area of each 4 mm core was captured with a Leica (DM500/13613205) microscope camera. Captured images were counted (400 tumor cells), and the labeling index (LI) for MCM6 and Ki-67 was calculated as the percentage of tumor cells with nuclear staining. This procedure was done independently by two pathologists. Also, the expression of MCM6 and Ki-67 was evaluated in epithelial breast tissue by the same method, using mammoplasty samples.

The information required for the 5-year follow-up of these patients and the status of their hormone receptors and HER2 were obtained from the Hospital Tumor bank’s database and their medical records, which were available at the pathology department archives. Using these data and the result of Ki-67 LI for each case, classification of different molecular subgroups according to St. Gallen criteria was done [21, 22]. This criterion was established in 2011 to offer a more convenient and feasible approach for the molecular classification of breast cancer. In brief, this clinicopathological criteria uses the expression status of estrogen and progesterone receptors (assessed by IHC), the overexpression and/or amplification of HER2, and the Ki-67 LI to classify breast cancers into four molecular subtypes [23]. The cut-off values for these markers are derived from the PAM50 classifications system [22].

### Statistical analysis

For statistical analysis of the results, we used IBM® SPSS® statistics version 23 (Armonk, USA). The distribution of variables was assessed by the Kolmogorov-Smirnov test. According to the non-normal distribution of both Ki-67 and MCM6 markers, we used non-parametric tests (such as Mann-Whitney and Kruskal-Wallis) for qualitative and Pearson regression for quantitative variables comparisons for further analyses. A *P*-value of < 0.05 was considered significant. We used the Bonferroni correction for the significant *P*-values of pairwise comparisons that we made between subgroups of a general group (such as luminal subtypes and overall histologic grade of tumors). To determine the optimum cut-off points and the diagnostic power of the variables, we used the receiver operating characteristic (ROC) curve and considered the sum of the squares of both sensitivity and specificity and the area under the curve (AUC) of them. For survival analysis, we used the Kaplan-Meier analysis method and variables with *P*-values of < 0.2 were included in a multivariable analysis by the cox regression model.

## Results

The mean age of patients was 49.11 years (range, 39 to 59 years). Among the investigated samples, 44.3% were luminal A, and 27.9% were luminal B subtypes. Besides, most samples in our study had an advanced form of the disease. The details of clinicopathologic features are provided in Table 1.

**Table 1:** Histopathologic descriptions of tumoral specimens

Variable	Frequencies (%)
Nuclear Grade	
I	2 (1.6)
II	48 (38.7)
III	74 (59.7)
Mitotic Grade	
I	51 (41.1)
II	27 (21.8)
III	46 (37.1)
Tubule Formation	
I	9 (7.3)
II	40 (32.3)
III	75 (60.5)
Overall Histologic Grade	
I	18 (14.5)
II	52 (41.9)
III	54 (43.5)
Molecular Subtypes	
Luminal A	54 (44.3)
Luminal B	34 (27.9)
HER2-enriched	12 (9.8)
Triple-Negative	22 (18.0)
Lymphovascular Invasion	
Negative	23 (18.5)
Positive	101 (81.5)
Lymph node Involvement	
Negative	46 (37.4)
Positive	77 (62.6)
Extra-nodal Extension	
Absent	26 (33.8)
Present	51 (66.2)
In-situ Carcinoma	
Negative	28 (22.6)
Positive	96 (77.4)
Estrogen Receptor	
Negative	35 (28.2)
Positive	89 (71.8)
Progesterone Receptor	
Negative	39 (31.5)
Positive	85 (68.5)
HER2/Neu	
Negative	95 (76.6)
Positive	29 (23.4)

### Expression of MCM6 and Ki-67 in tumoral and normal breast tissue

The median and inter-quartile range (IQR) of MCM6 were significantly higher in tumors and also normal breast samples in comparison to Ki-67 (Table 2).

**Table 2:** Comparison of marker’s expression status (median percentage of the positive cell) in normal and tumoral samples

Marker	Normal breast	Tumors	*P*-value
Ki-67 (median, IQR)	4% [3–10]	12 (8.25–17)	**< 0.001**
MCM6 (median, IQR)	20% (13–23.5)	29.75 (21.37–39)	**< 0.001**

### Comparison of MCM6 and Ki-67 in different histologic grades and hormone states of breast cancers

The expression level of MCM6 was significantly increased in higher grades (*P* < 0.001, Table 3). Therefore, MCM6 could discriminate different histologic grades of breast cancers (*P* = 0.004 between grade I-II and *P* < 0.001 between grade II-III). Interestingly, the correlation coefficient of the mitotic count with MCM6 and Ki-67 LI was 0.388 (*P* < 0.001) and 0.267 (*P* = 0.004), respectively. As a result, MCM6 LI could discriminate different mitotic categories of the Nottingham histologic score (score 1 mitoses: 23.8, score 2 mitoses: 30.5, and score 3 mitoses: 36.5, *P* < 0.001, Table 3). Also, the MCM6 LI exhibited a significant difference in different scores of tubule formation and nuclear pleomorphism (*P* < 0.001 for both, Table 3).

**Table 3.** Comparison of MCM6 and Ki-67 expression levels based on different histologic and prognostic features.

Variable	Ki-67 Median (IQR)	*P*-value	MCM6 Median (IQR)	*P*-value
Estrogen Receptor		**0.017**		**< 0.001**
Negative	17.0 (9.0–24.0)		38.5 (32.0–43.0)	
Positive	10.0 (8.0–16)		26.5 (20.5–34.0)	
Progesterone Receptor		**0.003**		**< 0.001**
Negative	19.0 (9.5–24.0)		37.0 (29.7–42.2)	
Positive	10.0 (8.0–19.0)		26.9 (20.5–33.5)	
HER2/Neu		0.339		0.286
Negative	11.0 (8.0–17.0)		28.5 (21.2–37.7)	
Positive	13.0 (9.0–20.0)		33.5 (24.0–41.0)	
Molecular Subtypes		**< 0.001**		**0.001**
Luminal A	9.0 (7.0–10.0)		24.0 (18.0–31.5)	
Luminal B	16.0 (14.0–20.7)		30.5 (23.6–44.3)	
HER2-enriched	15.0 (7.0–23.0)		39.0 (18.0–41.5)	
Triple Negative	19.0 (9.0–38.0)		38.5 (34.7–53.0)	
Lymphovascular Invasion		0.618		0.936
Negative	10.5 (7.2–18.7)		30.2 (17.2–46.1)	
Positive	12.0 (9.0–17.7)		30.5 (21.6–38.1)	
Lymph node Involvement		0.888		0.142
Negative	12.0 (8.0–20.0)		33.0 (20.5–45.0)	
Positive	12.0 (8.5–17.5)		27.0 (21.5–36.2)	
Extra-nodal Extension		0.841		0.124
Negative	11.5 (9.0–20.2)		31.0 (22.4–37.4)	
Positive	11.0 (7.5–17.0)		31.0 (22.0–37.5)	
In-situ Carcinoma		0.925		0.873
Negative	12.0 (7.0–19.5)		29.7 (15.7–42.6)	
Positive	12.0 (9.0–17.5)		30.5 (21.5–38.6)	
Nuclear Grade		0.071		**< 0.001**
I	–		–	
II	10.0 (7.5–16.0)		23.5 (17.0–30.5)	
III	13.0 (9.0–21.0)		34.0 (26.9–42.9)	
Mitotic Rate		0.068		**< 0.001**
I	10.0 (7.0–14.8)		23.8 (16.2–33.5)	
II	12.0 (9.0–16.0)		30.5 (23.1–41.5)	
III	15.0 (9.0–24.8)		36.5 (27.1–44.4)	
Tubule Formation		0.047		**0.002**
I	9.0 (5.0–10.0)		18.25 (9.5–29.87)	
II	12.0 (8.0–16.0)		26.0 (21.25–33.5)	
III	13.0 (9.0–24.0)		33.0 (23.0–42.5)	
Overall Grade		**0.006**		**< 0.001**
I	9.0 (5.0–10.0)		16.0 (10.2–26.0)	
II	11.0 (8.0–16.0)		25.5 (20.1–33.5)	
III	14.5 (9.0–22.5)		36.0 (28.5–45.0)	

Similarly, Ki-67 LI showed a significant statistical difference between different histologic grades of tumors (*P* < 0.001) and different scores of tubule formation (*P* = 0.006), but no significant correlations with nuclear pleomorphism (*P* = 0.071) and mitotic index (*P* = 0.068) were found (Table 3). As a result, unlike Ki-67, MCM6 could discriminate between 3 scores of mitotic indices. It should be noted that according to Bonferroni’s correction law, the significant *P*-value threshold in these types of pairwise comparisons was 0.017.

**Fig. 1:**
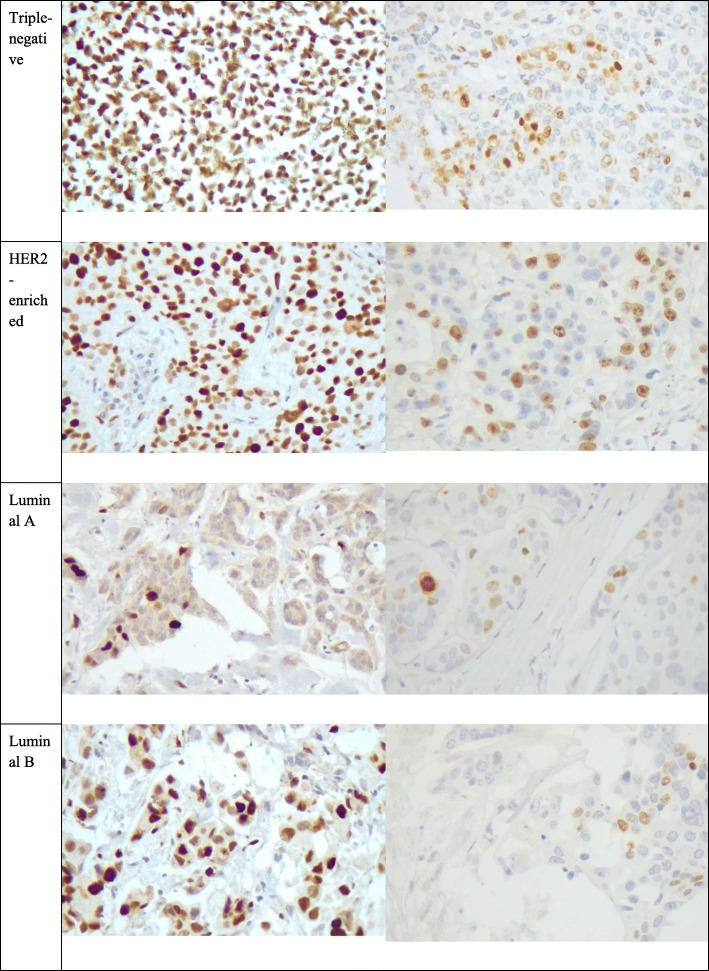
Comparison of MCM6 and Ki-67 staining of different molecular subtypes of breast cancer. Comparing luminal subtypes, the MCM staining level was significantly higher for the luminal B subtype. In addition, MCM6 and Ki-67 expression levels were both significantly higher in TNBC, compared with luminal A and B subtypes

The AUC in the ROC curves of these two markers confirms the aforementioned results. However, there was no optimum point for the sensitivity, specificity, and accuracy in these curves that could be used as an effective cut-off point value. Regarding HR and HER2 status, the median of both markers was significantly higher in HR-negative tumors (Table 3), but there were not any significant differences between these two markers according to the HER2 overexpression status (*P* = 0.339 for Ki-67, and *P* = 0.276 for MCM6, Fig. 1).

### Comparison of MCM6 and Ki-67 in different molecular subtypes of breast cancer

As depicted in Table 3, both Ki-67 and MCM6 scores were significantly varying in different molecular subtypes of breast cancer. Compared to the luminal A subtype, the median of MCM6 LI in the luminal B subtype was significantly higher (*P* = 0.01). Accordingly, when we considered the sum of both sensitivity and specificity, the optimal cut-off point for the discrimination of luminal A and luminal B subtypes was about 32%, with a sensitivity of 50% and a specificity of 77%. For Ki-67, we took 14% as the cut-off value, as validated by previous studies [22, 24].

Overall, both Ki-67 and MCM6 markers had a higher median in the TNBC than the luminal A and B subtypes (*P* = 0.016 and *P* < 0.001, respectively; Fig. 1). However, none of them could differentiate HER2-enriched subtype from the triple-negative and luminal subtypes (*P* = 0.433 and *P* = 0.118, respectively), which is most probably due to the small number of HER2-enriched samples in our study.

Furthermore, the comparison of MCM6 and Ki-67 in different histologic types, lymph node status, presence of extra-nodal extension, and the presence of in-situ carcinoma did not show any significant differences. The details of these comparisons are presented in Table 3.

### Prognostic values of MCM6

We followed 112 patients for a median of 62.46 months. Eighteen cases died due to breast cancer, and 24 cases showed recurrences of the disease. No significant correlation was found between MCM6 and Ki-67 expression and the overall survival (OS; for MCM6, hazard ratio [HR] = 1.996, 95% confidence interval [CI] = 0.474–8.401, *P* = 0.346; and for Ki-67, HR = 0.958, 95%CI = 0.193–4.755, *P* = 0.959) and disease-free survival (DFS).

## Discussion

Ki-67, as a well-known proliferative marker, is suggested as a valuable predictor of survival, recurrence, and aggressiveness of breast cancer [25, 26]. Besides, many studies have investigated the associations between Ki-67 levels and tumor’s grade, stage, lymph node involvement, and estrogen receptor (ER) status [25]. The stratification of breast tumors to different molecular subtypes (according to the hormone receptor, HER2, and Ki-67 expression) and the usefulness of Ki-67 as a tool for selecting systemic treatment for early-stage breast cancers is established [25]. However, Ki-67 faces some limitations. For instance, it is not expressed in cells that have the potential to enter the G1 phase of the cell cycle, which will pose a risk of the misidentification of tumoral cells [27]. Besides, its functions are not well understood, and more importantly, it has no generally accepted cut-off values [28].

MCM proteins have key roles in the regulation of DNA replication in eukaryotes. MCM family members have some superiorities over Ki-67. They can be detected in cells that are in the resting phase of the cell cycle but still have the replication competency. Also, their expression is stable during the cell cycle [7]. As a result, compared with Ki-67, a greater number of proliferative cells in different types of neoplasms would be identified by MCM [29]. Also, in normal breast terminal duct-lobular units (TDLU), levels of MCM expression are higher than Ki-67 and are consistent with the high proportion of mammary epithelial cells residing in a licensed MCM expressing but the non-proliferating state [5, 7, 18].

In this study, we aimed to reveal whether the MCM6 expression score can be interpreted as a proliferative marker and be used as an alternative to Ki-67 in differentiating molecular subtypes and also histologic grades of breast cancer. Therefore, we compared the expression of Ki-67 and MCM6 in 124 breast cancer samples with different grades and molecular subtypes.

In both normal and tumoral breast tissues, MCM6 had significantly higher levels of expression than Ki-67. The median of MCM6 and Ki-67 nuclear staining scores were significantly higher in tumors than in normal breast tissues. Hence, the influence of these markers on the carcinogenesis of breast tumors can be inferred. MCM6 can differentiate between histologic grades of invasive ductal carcinoma (*P* < 0.001) with a meaningful correlation with mitotic figures, which is also stronger than that of Ki-67. Thereby, it seems that MCM6 is capable of more precise classification of breast tumors regarding histologic and mitotic grades. Both Ki-67 and MCM6 revealed an association with ER status. HR negative tumors had higher MCM6 and Ki-67 expression.

The most important finding is that both MCM6 and Ki-67 can discriminate between luminal A and B molecular subtypes and between HR-positive and TNBC, which has a critical role in selecting the therapeutic strategies. As we have mentioned previously, we chose the cut-off point of 32% for the discrimination between the luminal A and B subtypes. We found no significant association between the expression levels of these two biomarkers and prognostic factors (such as lymphovascular invasion, lymph node involvement, tumor size, OS, and DFS), which is most probably due to the small number of assessed cases.

Many studies have evaluated the expression of various members of the MCM family besides other prognostic markers (such as P53) in several types of malignancies. For example, the upregulation of MCM gene expression in uterine cervical cancers is noted and reaffirms MCM as a proliferative marker in the DNA replication pathway, whereby the proliferation of dysplastic and cancer cells becomes increasingly dysregulated and proposed these markers as a valuable screening tool in detecting pre-cancerous cervical lesions [30].

In recent years, several studies have focused on the expression of various members of the MCM family in breast cancers and have reported relatively comparable results. In a study by Cobanoglu et al., the expression of MCM2 had a significant association with the histologic grade of breast carcinoma and the cell proliferation capacity (indicated by Ki-67). In addition, a negative correlation between MCM2 or Ki-67 expression and ER expression was reported. They observed no significant association between MCM2 or Ki-67 expression and patients’ age, tumor size, lymph node status, clinical stage, and menopausal status [31]. Our results for the expression of MCM6 and Ki-67 are in concordance with the MCM2 expression in this study.

Another study showed that after a median follow-up of 5.3 years, fluorescence in situ hybridization (FISH) assessed MCM2 LI is not predictive of disease recurrence. However, they have reported MCM2 as a useful marker for distinguishing the aggressive-type *HER2*-amplified breast carcinomas (with high malignancy grade) from HR-negative subtypes [32]. They also proposed that despite the acceptable correlation between MCM2 and Ki-67, MCM2 protein can be a superior proliferative marker in discriminating different histologic grades of breast cancer [33]. In our study, we observed comparable results for MCM6 and its meaningful correlations with tumor grade, hormone receptor status, and molecular subtypes.

In a study designed by Issac et al., MCM2, MCM4, and MCM6 were assessed at the level of mRNA transcription and protein expression in breast cancers. They concluded that these markers can especially help to differentiate between luminal A and luminal B subtypes. Also, they found a meaningful correlation between these markers and Ki-67 and the histologic grade. Low expression of these markers was associated with an increased probability of relapse-free survival [17]. To the best of our knowledge, this study is the only one that has evaluated MCM6 in breast cancers. Our results are concordant with the findings of this study.

MCM family members are one of the proliferative markers, and their role in the determination of proliferative activity and their relationship with prognostic and therapeutic factors, even in a stronger power rather to PCNA and Ki-67, is confirmed [34]. MCM proteins are highly expressed in malignant human cancer cells and pre-cancerous cells undergoing malignant transformation, but not in the differentiated somatic cells. Therefore, these proteins are ideal diagnostic markers for cancer and serve as promising targets for anti-cancer drug development [6, 35].

There are limitations to this study. The retrospective design hampers reaching definite and casual associations. Until now, there are no valid and standardized methods for the assessment of MCM6 expression level. The low prevalence of HER2-enriched tumors in our samples was a major obstacle for MCM6 to differentiate them from other molecular subtypes. Lastly, the optimal cut-off points for MCM6 LI to differentiate between luminal A and luminal B subtypes led to a sensitivity of 50%, which generally is not acceptable for diagnostic tests. Larger prospective studies are warranted to further evaluate the importance of MCM6 as a diagnostic and prognostic marker for breast cancer.

In conclusion, our data support that MCM6 is a superior discriminator of tumor grade by better capturing the scoring differences in all three parameters of the Nottingham Score better than Ki-67. In addition, it can be taken as an alternative marker for Ki-67 in the classification of breast tumors into different molecular subtypes, especially luminal ones. Differentiating luminal A and B cancers remains a significant clinical question since it impacts therapeutic decisions. In this study, we tried to determine an appropriate cut-off value point for differentiating between these subtypes. Maybe in the future, more detailed data regarding these issues can help establish a more precise and accurate cut-off point with higher specificity and sensitivity. Surely the suitability of this marker for routine clinical use instead of Ki-67 has more unknown aspects to be investigated in the future.

## Abbreviations

MCM6: Minichromosome maintenance protein 6
HR: Hormone receptor
HER2: Human epidermal growth factor receptor 2
FFPE: Formalin-fixed paraffin-embedded samples
IHC: Immunohistochemistry
H&E: Hematoxylin and eosin
LI: Labeling index
ROC: Receiver operating characteristic
AUC: Area under the curve
IQR: Inter-quartile range
OS: Overall survival
HR: Hazard ratio
CI: Confidence interval
DFS: Disease-free survival
ER: Estrogen receptor
TDLU: Terminal duct-lobular units
FISH: Fluorescence in situ hybridization
